# Changes in pain following an interaction period of resistance training and green tea extract consumption in sedentary hypertensive women: impact of blood pressure swings

**DOI:** 10.1186/s41043-019-0188-y

**Published:** 2019-10-31

**Authors:** Hamid Arazi, Behzad Taati, Jalal Kheirkhah, Samaneh Ramezanpour

**Affiliations:** 1 Department of Exercise Physiology, Faculty of Sport Sciences, University of Guilan, P.O.Box: 1438, Rasht, Iran; 2 Department of Cardiology, Healthy Heart Research Center, Heshmat Hospital, School of Medicine, Guilan University of Medical Sciences, Rasht, Iran

**Keywords:** Resistance training, Catechins, Hypoalgesia, Pain threshold, Pain perception, Blood pressure

## Abstract

**Background:**

Changes in blood pressure (BP) may affect pain. However, the interaction effect of resistance training and green tea on BP and pain has not been studied. The primary aim of this study was to evaluate the impact of resistance training and green tea extract (GTE) on pain variables in hypertensive patients. Secondary aim included determining the effects of BP alterations on pain responses.

**Methods:**

In a randomized, double-blind, placebo-controlled study, 30 middle-aged sedentary women were randomly divided into resistance training and green tea extract (GR, *n* = 8), resistance training (R, *n* = 8), green tea (G, *n* = 7), and control groups (C, *n* = 7). The study period consisted of 3 weeks of GTE (~ 245 mg total polyphenols) consumption twice a day followed by 6 weeks of interaction with resistance training. GR and R groups performed two circuits of training with ten repetitions at 50% of 1RM 2 days a week while other two groups had no any regular exercise training. R and C groups also received placebo capsules (maltodextrin) with the same timing. Pain threshold and perception, BP, and heart rate were recorded following the first and last session of training at rest and 5th and 15th minute.

**Results:**

Pain perception of training groups after the last session was significantly higher than control conditions, and at this time, the magnitude of BP responses was lower in training groups. In proportion to pain threshold, there were no significant differences between groups.

**Conclusion:**

It seems that training-induced hypotension can alter pain perception in hypertensive women through changes in baroreceptor activation.

## Implications

- Training-induced adaptations exhibit an increase in pain perception through reducing systolic blood pressure.
- There is a trend to go up in pain threshold following resistance exercise.
- Blood pressure is associated negatively with pain perception.
- Nine weeks of green tea extract ingestion does not make significant differences in pain responses compared to resistance training alone.

## Background

In recent years, the number of studies investigating the health-related properties of green tea (GT), including neuroprotective effects [1] have risen dramatically. GT and its extract (GTE) and also its isolated constituents are associated with improving cardiovascular and metabolic health [2]. The results of studies show that GT has favorable effects on the brain and nervous system [3, 4]. Previous evidence has indicated that GTE can reverse the lipopolysaccharide-induced hyperalgesia in mice [5]. Renno et al. [6] examined the effect of GT in unilateral chronic constriction injury (CCI) to the rat sciatic nerve, and they observed a significant decrease in the behavioral mechanical hyperalgesia in GT groups.

Furthermore, physical activity can affect the nervous system, and some studies have reported an attenuation of pain following an acute exercise in healthy subjects, which has been called exercise-induced hypoalgesia (EIH) [7–9]. The mechanisms responsible for EIH are still not entirely clear and it is most likely multifactorial. Results suggest an interaction between pain regulatory and cardiovascular system. The brain stem nuclei are associated with blood pressure (BP) and pain modulatory system. Thus, the hypertension-hypoalgesia hypothesis has been proposed because BP naturally increases during the exercise. Based on this hypothesis, higher BP can lead to baroreceptor activation and consequently decreases in pain sensitivity [10, 11].

The results of several studies indicate that GT and epigallocatechin-3-gallate (EGCG) can induce favorable effects on BP in hypertensive rats [12, 13] and hypertensive women [14]. Our previous results [14] showed that short-term ingestion of GTE did not influence systolic and diastolic BP and heart rate (HR), but provoked a favorable effect on mean BP and rate pressure product responses.

Taken together, there is some evidence indicating that pain and cardiovascular responses are altered following exercise, but our data is limited in relation to resistance training effects in hypertensive individuals. Therefore, the primary purpose of this study was to examine whether a period of GTE consumption and resistance training produce alterations in pain threshold and perception in hypertensive women. In addition, it has been shown that hypertensive individuals exhibit reduced pain sensitivity compared with normotensive individuals [15]. Thus, our secondary purpose was to examine whether these effects are along with BP alterations.

## Methods

### Participants

In this study which was approved by the Ethics Committee of the Department of Sports Sciences, University of Guilan, 49 sedentary hypertensive women, ages 35–55 years, were recruited from the Cardiovascular Hospital of Guilan and signed written informed consent for data recording (Table 1).

**Table 1:** Mean (SD) of physical and hemodynamic characteristics of each group

	GR (*n* = 8)	R (*n* = 8)	G (*n* = 7)	C (*n* = 7)	*P* value
Age (year)	46.12 (5.4)	45.12 (6.7)	49.57 (6.85)	46.14 (7.01)	0.592
Height (cm)	168 (1.02)	165.62 (1.01)	168.86 (0.03)	167.86 (1.13)	0.067
Weight (kg)	68 (9.14)	66.62 (5.09)	70.85 (8.35)	71 (8.52)	0.645
BMI (kg/m^2^)	24.10 (3.31)	24.27 (1.69)	24.83 (2.65)	25.18 (2.81)	0.857
Body fat (%)	26.76 ± (4.36)	27.61 (3.88)	27.71 (2.03)	27.20 (4.3)	0.977
Systolic BP (mmHg)	133.12 (3.72)	136.87 (5.93)	130.71 (4.49)	137.14 (6.98)	0.685
Diastolic BP (mmHg)	85.62 (3.20)	85.62 (5.62)	80.71 (4.49)	87.14 (6.36)	0.296
Heart rate (b.p.m)	79.50 (4.65)	78 (9.13)	70.28 (7.69)	74.28 (6.79)	0.156

*GR* green tea extract and resistance training group, *R* resistance training group, *G* green tea extract group, *C* control group, *BMI* body mass index, *BP* blood pressure

**Fig. 1:**
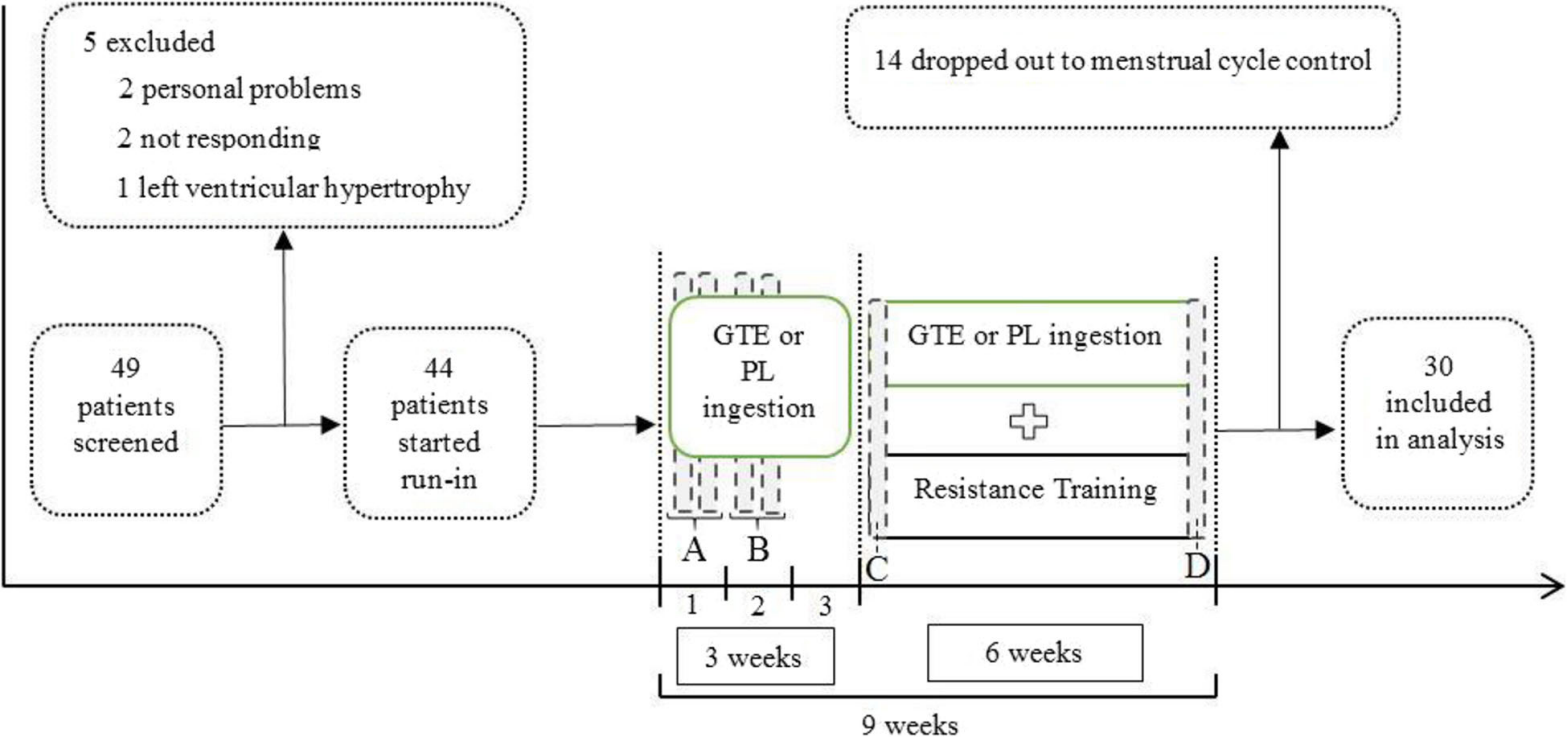
Schematic representation of study design. (**a**) Familiarization sessions. (**b**) 1RM test sessions. (**c**) Measurements after the first session. (**d**) Measurements after the last session. GTE, green tea extract; PL, placebo

Exclusion criteria included any musculoskeletal or renal disease, regular activity before the trial for a minimum period of 6 months, pregnancy, menopause, or any disturbance in the menstrual cycle [16], and the use of any pain medications. The participants received a resting 12-lead electrocardiogram (ECG) and a cardiac stress test in order to ensure the cardiovascular health. After initial drop out, the remaining patients (*n* = 44) were randomly divided into four groups: resistance training and green tea extract (GR), resistance training (R), green tea (G), and control group (C). Fourteen patients were excluded from the study during the final stage and 30 patients completed the trial (Fig. 1).

### Menstrual cycle control

Given the possible effects of BP on pain threshold and its perception, the menstrual cycle was controlled as a factor affecting BP. There are conflicting results about the effects of menstrual cycle on BP. A higher BP during the early follicular than the luteal phase [17] or no difference between phases [18] has been reported. The participants completed menstrual cycle questionnaire [19] 48 h before the trial and after the final session of training. Ultimately and after the post-test measurements, patients in the early follicular period were excluded from the study (14 patients).

### Diet and capsules content

All the participants completed food diary of 1 week prior to pre-tests measurements. Then, they received recommendations to reduce or increase energy intake and were asked to maintain their diet until the end of the study. They also completed food diary of 1 week before the first and final session of resistance training. Table 2 shows that groups in the approximate amount of energy, sodium, and potassium intake were not significantly different from each other during the study period.

**Table 2:** Mean (SD) of dietary intake of each group

	GR	R	G	C	*P* value
Energy intake (Cal)	2350.06 (102.66)	2430.56 (188.94)	2369.71 (160.49)	2341.14 (136.37)	0.649
Sodium (g)	2.84 (0.64)	2.81 (0.51)	2.87 (0.69)	2.74 (0.6)	0.982
Potassium (g)	3.37 (0.86)	3.77 (0.99)	3.54 (1.24)	4.09 (0.86)	0.550

*GR* green tea extract and resistance training group, *R* resistance training group, *G* green tea extract group, *C* control group

This study was a randomized double-blind, placebo-controlled trial consisting of a 9-week GTE consumption period. Patients ingested two capsules (500 mg) [2] containing either GTE (~ 245 mg total polyphenols,~ 75 mg EGCG, ~ 25 mg caffeine) or placebo (~ 490 mg maltodextrin) after lunch and dinner every day besides their usual medication. The capsules were prepared in the same formation and color and were also scorched with GT to minimize differences between them. Dose, type, and the hour of medications did not change during the study.

The subjects were asked to refrain from GT intake (other sources) as well as to reduce the consumption of black tea. They were not taking any capsule in the trial day and were instructed to avoid consumption of caffeine-containing items (tea, coffee, chocolate, and energy drinks) for at least 3 h before the intervention.

### Familiarization, 1RM testing, and training protocol

All the subjects were familiar with resistance training machines and devices, correct technique, the normal range of motion, and suitable breathing during two separate days. In these sessions, they performed two sets of 15 repetitions without load. The ten-repetition maximal (10RM) test was conducted 72 h after familiarizations days and during two next sessions. 1RM records were calculated using the equation provided by Brzycki [20].

$$ 1\mathrm{RM}=\frac{\mathrm{weight}\ \left(\mathrm{kg}\right)}{1.0278-\left(0.0278\times \mathrm{number}\ \mathrm{of}\ \right)} $$

The exercise protocol was 6 weeks of circuit resistance training with resistance machines in which performed 2 days a week in the afternoon (4–6 P.M). Training sessions were carried out following order after 10 min of warm-up consisted of walking and static stretching: seated bench press, seated leg press, lat pull down, seated knee extension, seated biceps curl, and leg curl. In these sessions, they performed two sets of ten repetitions with the intensity of 50% of 1RM. Rest intervals between the sets and circuits were 2 min.

### Measurements and experimental procedure

BP (standard mercury sphygmomanometer; ALP K2; 300-V-EU; Japan) was assessed via auscultation of the first and fifth Korotkoff sounds for systolic and diastolic BP, respectively. HR was also measured by an automatic HR monitor (Beurer; PM80; Germany).

The pain stimulus was created by pressure (1 kgf) being applied to the middle digit of the left hand with an algometer [7, 21]. The participants inserted their finger into the chute of the algometer and pain threshold was recorded by a stopwatch (Q & Q; HS43), as the point beginning pressure to the point at which noxious stimulation is first perceived to be painful. Also, pain perception was obtained by a vertical descending visual numeric pain scale [22]. This scale has 11 numbers from 0 to 10 with visual descriptors which have been attached to the numbers and represent the continuum from no pain (score 0) at the bottom to maximum levels of pain (score 10). The pain scale was placed in front of the participants, and they determined a score for their pain immediately after threshold test. This procedure had already been done several times and during separate days. Therefore, the patients were familiar with the noxious stimulation and pain perception scores.

The participants were also familiarized with the measurement environment, procedure, and equipment for several days to reduce their stress. All the procedures were performed between 3:30 and 6:30 P.M. During the preliminary session, patients received their GTE or placebo capsules in a randomized order and were asked to consume them for 3 weeks to ensure there is no problem in taking the capsules. Anthropometric measurements including height, weight, and body fat percent (skinfold thickness; Lafayette Instrument Co, 01127A, USA) were assessed during the first familiarization session. In the third and fourth sessions, the 1RM test of mentioned exercises was conducted, and the training period (6 weeks) begun days later. At the beginning of the first training session and after a minimum of 5 min of seated rest, BP and HR were measured three times with 5-min intervals and were averaged. Then, baseline pain threshold and perception were obtained. The variables were also recorded at the 5 and 15 time points during the recovery period of the first and last session of resistance training.

### Data analysis

The two-way repeated measure of ANOVA (4 trials × 6 times) followed by post hoc Bonferroni test was used to evaluate intragroup differences. A *P* value of *P* < .05 was considered as significant level, and the SPSS software (v. 20®, Inc. Chicago, IL) for the Windows computer was used to analyze the data.

## Results

No statistical differences were found among the groups regarding anthropometric and hemodynamic characteristics (Table 1). Pain threshold (4.41 ± 3.09), pain perception (0.009 ± 0.21), systolic BP (0.95 ± 1.68), diastolic BP (0.99 ± 2.58), and HR (− 1.06 ± 0.74) were not changed in the C group.

**Fig. 2:**
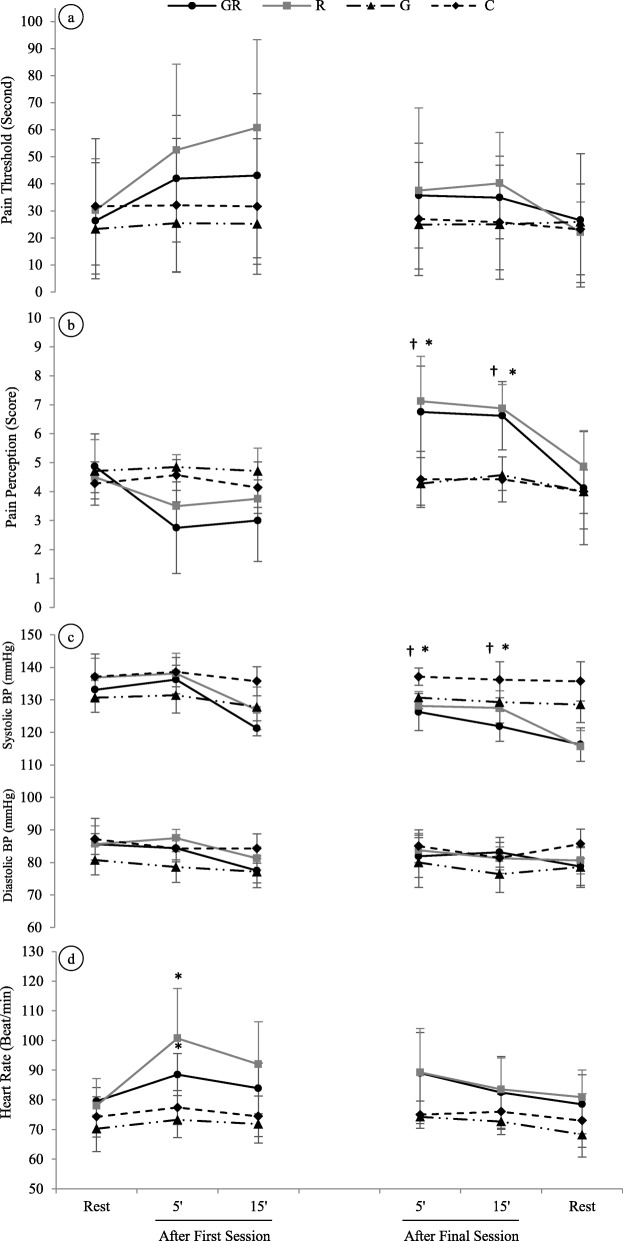
Changes in hemodynamic and pain measurements after the first and final session of resistance training in exercise and green tea extract (GR), resistance training (R), green tea extract (G), and control (C) groups. **a** Changes in pain threshold responses after exercise and quiet rest. **b** Changes in pain perception responses after exercise and quiet rest; *P* < 0.05 between C and G* with RG, and ^†^ with R. **c** Changes in blood pressure responses after exercise and quiet rest; *P < 0.01 between GR and C; ^†^*P* < 0.05 between R and C. **d** Changes in heart rate responses after exercise and quiet rest; *P < 0.05 vs rest

Figure 2a shows the results of pain threshold at rest and minute 5 and 15. Pain threshold of training groups (GR and R) tended to go up following exercise in comparison to control groups (C and G), but there were no significant differences between groups after either first or last session of the resistance training.

As shown in Fig. 2b, pain perception was increased in training groups compared with control groups at the 5th minute (GR vs C, *P* = 0.018; GR vs G, *P* = 0.011; R vs C, *P* = 0.005; and R vs G, *P* = 0.003) and the 15th minute (GR vs C, *P* = 0.001; GR vs G, *P* = 0.002; R vs C, *P* < 0.01; and R vs G, *P* < 0.01) after last session. The magnitude of pain perception also was lower in training groups compared to control groups after the first session, but these differences did not reach significance.

The results for systolic and diastolic BP are illustrated in Fig. 2c. After the first session, systolic BP was not found to differ between groups, although it was decreased in the GR group at the 15th minute. Systolic BP also was found to be decreased significantly between the GR and C groups (*P* < 0.01) and the R and C groups (*P* = 0.01) after the last session.

After the first session, HR of GR and R groups at the fifth minute was higher compared with resting HR (− 9 ± 4.27 and − 22.75 ± 12.83 for GR and R groups, respectively). Despite the higher magnitude of HR in training groups following exercise, these changes were not found to differ in comparison to the control groups. The results for HR are indicated in Fig. 2d.

## Discussion

This study investigated the interaction effect of resistance training and GTE consumption on the responses of pain threshold and perception in hypertensive women without any change in their usual lifestyle. We also aimed to assess the changes in BP and HR during a two-time exposure to a noxious pressure stimulus before and following 6 weeks of resistance training. The participants had a resting BP higher than normal range, and consequently, they were under physician’s care. The main findings of this study included the following: (1) the pain perception responses of training groups (GR and R) after the first session of training were lower than that of the control groups (G and C), while it was significantly increased after the last session and at these time points and (2) the magnitude of systolic BP responses in training groups were lower than that in the C group. Thus, it seems that the changes in systolic BP have a significant effect on pain perception only after the training period while there was no significant effect after the first session. (3) There were also no significant differences between the GR and R groups in terms of pain variables. In other words, 9 weeks of GTE ingestion did not make significant differences in pain responses of the GR group compared with the R group.

Pain stimulus should not cause tissue damage or injury for acute pain measurement. Skin and body temperature will change during physical activity depending on the duration and intensity of exercise. Thus, thermal stimulus (heat or cold) are not suitable for the measurement of the changes in pain variables after exercise. Algometer is a simple, practical, and reliable device [21] which can measure the changes in pain responses by creating a mechanical stimulus (pressure) on the subject’s finger.

In this study, pain perception of training groups was significantly higher than that of the control groups after the last session. At the same time points, the training groups had a lower systolic BP compared to the C condition. These results propose that training-induced adaptations in the training groups make an increase in pain perception of patients through reducing systolic BP, and consequently, they described induced pain by higher scores.

Koltyn and Arbogast [7] reported that resistance training (70% of 1RM) increased pain threshold at minute 5 after exercise while pain perception was decreased. In this study, the values returned to baseline after 15 min. As shown in Fig. 2a, the pain threshold of the training groups tended to go up following exercise, but these changes did not reach significance. Thus, the pattern of pain threshold responses in the present study is different from that reported by the mentioned investigators [7]. The inconsistency between studies may partially account for participants condition (i.e., hypertensive patients vs healthy subjects), different exercise protocols (i.e., different exercise intensities vs different exercise durations), and training-induced adaptations (i.e., 6 weeks of resistance training vs single bout). In line with our results, Bartholomew and colleagues [23] examined the influence of 20 min of circuit resistance training or stationary cycling on pain threshold and tolerance. Their findings indicated that pain tolerance was changed compared to the control condition, but pain threshold did not change significantly following exercise. The certain reasons for these changes in the pain perception of the training groups are not completely understood, but it seems that there is an inverse relationship between pain perception and BP as reported in former studies [24, 25]. The present findings are added to the small database investigating the relationship between BP and EIH in women and confirm that BP is associated negatively with pain perception and positively with pain thresholds, as reported by Ghione [10].

Based on the present results, the systolic BP of the training groups was lower in comparison to the C group after the final session, but the differences were not significant after the first session. The HR of participants had a considerable increase after the first session compared to the resting values, but no significant difference existed after the last session. These findings suggest that low-intensity resistance training can cause the cardiovascular adaptations in hypertensive women, as BP responses were decreased after 6 weeks. The current findings are also consistent with our previous research where we found that 3 weeks of GTE consumption had no significant effect on the magnitude of reducing systolic and diastolic BP following a session of low-intensity resistance training [14].

Previous studies have noted that two potential mechanisms may explain the BP-induced hypoalgesia, including baroreceptor activation and endogenous opioids. It has been proposed that increased BP may produce a hypoalgesic effect through activation of baroreceptor afferents [24]. Given that systolic BP was reduced after the training period in the present study, it seems that this reduction in BP is, at least in part, the possible mechanism for the increase in pain perception. However, more release of endogenous opioids can also affect pain perception. Based on the reported findings, hypertensive individuals exhibit higher levels of circulating endorphins and diminished sensitivity to painful stimulus [26]. Hoffman and Thoren [27] reported that when baseline BP values are changed due to physiologic or pathophysiologic stimulation, opioid system is activated and released endorphin in this situation which is related to exercise-induced hypoalgesia. In relation to effects of endorphin on pain sensitivity, the most studies have used aerobic exercise and few studies have been done with resistance exercise. Also, the results of one study using isometric exercise in men showed that exercise may induce hypoalgesia by an arterial baroreceptor inhibition mechanism [11]. Further research is needed to understand mechanism/mechanisms of resistance exercise-induced hypoalgesia.

Studies have reported that GT ingestion has a favorable effect on nervous system and pain stimulation factors [3, 5, 6]. It has been reported that in in vitro model, the rats with CCI to the sciatic nerve showed a decrease in pain variables as a result of GT intake [6]. In that study, the GT groups (three groups with different timing of GT intake) had a lower pain behavior compared to the control condition, and the authors suggested also that GT intake can produce significant neuroprotective effects [6]. In the present research, GTE consumption for 9 weeks in the GR and G groups in comparison to low-intensity resistance training had no effect on pain threshold and perception in hypertensive women, and the combination of these two factors did not produce significant interaction effect on pain responses. Given the components of GT, its consumption for a longer period or with higher doses of catechins may be effective in relieving pain. Therefore, it seems that further research is needed in this area.

In summary, these findings suggest that resistance training can alter pain sensitivity in hypertensive women through significant reductions in BP responses following exercise. We did not measure endogenous opioid (i.e., endorphin) in the current research, but it seems that altered baroreceptor activation is one of the more possible mechanisms for training-induced hypoalgesia.

This study has some limitations. Ten patients were taking antihypertensive drugs (beta blocker) which dose and times of medication were the same in all investigation period. However, it has been suggested that the administration of these drugs has never demonstrated a significant reduction of hypoalgesia [28]. Although the amounts of tea and other dietary intake were monitored by food diary, the exact amounts of catechin intake were not controlled separately.

## Data Availability

The datasets during and/or analyzed during the current study are available from the corresponding author on reasonable request.

## Abbreviations

1RM: 1-repetition maximal
BP: Blood pressure
C: Control group
CCI: Chronic constriction injury
ECG: Electrocardiogram
EGCG: Epigallocatechin-3-gallate
EIH: Exercise-induced hypoalgesia
G: Green tea group
GR: Resistance training and green tea extract group
GT: Green tea
GTE: Green tea extract
HR: Heart rate
R: Resistance training group
